# Carbon Nanomaterials for Therapy, Diagnosis and Biosensing

**DOI:** 10.3390/nano12091597

**Published:** 2022-05-09

**Authors:** Antonino Mazzaglia, Anna Piperno

**Affiliations:** 1 CNR-ISMN, Istituto per lo Studio dei Materiali Nanostrutturati, URT of Messina, Department of Chemical, Biological, Pharmaceutical and Environmental Sciences, University of Messina, V. le F. Stagno d’Alcontres 31, 98166 Messina, Italy; 2 Department of Chemical, Biological, Pharmaceutical and Environmental Sciences, University of Messina, V. le F. Stagno d’Alcontres 31, 98166 Messina, Italy

In carbon nanomaterial design, the fine-tuning of their functionalities and physicochemical properties has increased their potential for therapeutic, diagnostic and biosensing applications [1,2,3]. In this Special Issue, articles or mini reviews on nanoplatforms originating from the synergistic combination of carbon-based nanomaterials (i.e., nanotubes, graphene, graphene oxide, carbon quantum dots, nanodiamond, etc.) and various functional molecules such as drugs, natural compounds, biomolecules, polymers, metal nanoparticles and macrocycles relevant in drug delivery, in multi-targeted therapy, in theragnostics, as scaffolds in tissue engineering and as a sensing material, have been selected for publication. 

Trapani et al. investigated the ability of multiwalled carbon nanotubes (MWCNTs) covalently modified with polyamine chains of various length (ethylenediamine (EDA) and tetraethylenepentamine (EPA)) to induce the J-aggregation of meso-tetrakis(4-sulfonatophenyl)porphyrin (TPPS) in different experimental conditions. The authors reported that, in mild acidic conditions, TPPS porphyrin easily self-assembles into J-aggregates, showing peculiar extinction bands in the visible region (λ ≅ 493 nm) and in the therapeutic window (λ ≅ 710 nm), together with an emission band in the red spectral region. The results of this study describe the experimental conditions in which to obtain stable TPPS J-aggregates in medium mimicking physiological conditions for a stimuli-responsive therapeutic action upon irradiation on their extinction bands and fluorescence probes in cellular environments [4]. 

In the design of diagnostic nanoplatforms, aiming to improve the surface-enhanced Raman spectroscopy (SERS) effect, Neri et al. proposed a new graphene/gold nanocomposite composed of gold nanoparticles (AuNPs), produced by pulsed laser ablation in liquids (PLAL), and a nitrogen-doped graphene platform (G-NH2) obtained by direct delamination and chemical functionalization of graphite flakes with 4-methyl-2-p-nitrophenyl oxazolone, followed by the reduction of p-nitrophenyl groups. This approach allowed the authors to study SERS properties of graphene loaded with pure AuNPs without the influence of capping agents, surfactants, or salt produced in the chemical reduction of gold ions. The SERS platform was tested for its ability to detect Rhodamine 6G and Dopamine as molecular probes at a concentration around 1 µM. The platform showed good stability and the ability to reproduce Raman signals without degradation although its sensitivity was low. Overall, the feasibility of the proposed method opens up the field to further research on improving the detection limits of molecular probes interacting with loaded AuNPs [5]. 

Nowadays, new therapeutic approaches using carbon nanomaterials have become very attractive. In this scenario, Pennetta et al. investigated the formation of Doxorubicin (DOX) nano-conveyors as a stacked drug-delivery system for application in cancer treatment. The innovative nanoplatform was obtained by functionalizing single- and multi-walled carbon nanotubes (CNT and MWCNT, respectively) by cycloaddition reaction between carbon nanotubes and a pyrrole-derived compound. Two different adducts between CNT and pyrrole polypropylene glycol (PPGP) were prepared: the supramolecular adduct (CNT/PPGP_s_) and the covalent one (CNT/PPGP_c_). The supramolecular interactions were studied on the basis of molecular dynamics simulations, and by monitoring the emission and the absorption spectra of DOX. The biological studies revealed that two of the synthesized nanoplatforms are effectively able to obtain DOX within A549 and M14 cell lines and to enhance the cell mortality at a much lower effective dose of DOX. This work paves the way for the facile functionalization of carbon nanotubes by exploiting the “pyrrole methodology” for the development of novel technological carbon-based drug-delivery systems [6].

One of the main widespread concerns regarding using carbon-based materials as alternative nanobiomaterials for cancer therapy is their inherent cytotoxicity, which remains debated, with studies demonstrating contradictory results. Garriga et al. investigated the in vitro toxicity of various carbon nanomaterials in human epithelial colorectal adenocarcinoma (Caco-2) cells and human breast adenocarcinoma (MCF-7) cells. Carbon nanohorns (CNH), carbon nanotubes (CNT), carbon nanoplatelets (CNP), graphene oxide (GO), reduced graphene oxide (GO) and nanodiamonds (ND) were systematically evaluated and compared using Pluronic F-127 as a dispersant agent. Carbon nanomaterial exposure affected the cell viability in the following order: CNP < CNH < RGO < CNT < GO < ND, with a pronounced effect on the more rapidly dividing Caco-2 cells. Hydrophobicity and morphological features are the main causes of decreases in cell viability, enhanced levels of ROS (radical oxygen species) and apoptosis/necrosis. In this study, ND showed low toxicity thanks to a lack of ROS levels and was efficient in the loading of hydrophilic drugs, such as DOX, by assembling in the ND surface or within the pores. On the other hand, this study evidenced the low toxicity of CNT and RGO, and the high camptothecin (CPT) loading because of the strong π–π stacking interactions. Altogether, despite the various obstacles that still have to be overcome before considering carbon nanomaterials suitable as drug carriers (i.e., the potential long-term toxicity), this study highlighted a screening and risk-to-benefit assessment and, together with drug-loading efficiency studies, is fundamental to the development of advanced multi-functional carbon nanomaterials for cancer theragnostic applications [7]. 

Nanodiamonds with detonation origins were investigated by Claveau et al. as delivery systems for anti-cancer therapy in vivo models. The authors studied the ability of cationic hydrogenated detonation nanodiamonds to carry active small interfering RNA (siRNA) in a mice model of Ewing sarcoma, which is bone cancer of young adults due to the *EWS-FLI1* junction oncogene in the majority of patients. Labeled nanodiamonds obtained using radioactive tritium gas instead of hydrogen gas allowed the authors to investigate the trafficking of nanodiamonds throughout mouse organs and their excretion as urine and feces. Moreover, the ability of siRNA to inhibit the expression of the oncogene *EWS-FLI1* in tumor-xenografted mice was demonstrated. Overall, this study represents a substantial step towards the use of ultra-small solid nanoparticles for the delivery of nucleic acid in vivo [8].

In the framework of carbon nanomaterial development for therapeutic purposes, Lee et al. reported a new type of carbon dot (CDOT) nanoparticle as a new antiplatelet agent. The inhibition of platelet activation is considered a potential therapeutic approach for the treatment of arterial thrombotic diseases; therefore, maintaining platelets in their inactive state has gained much attention. CDOT could actively inhibit human platelet activation by suppressing some crucial mechanisms (e.g., PKC activation, and Akt, JNK1/2 and p38 MAPK phosphorylation) with no in vitro cytotoxicity. This in vivo study revealed that the CDOTs had an antithrombotic effect on the ADP-induced pulmonary thromboembolic mice model by reducing mortality and by preserving the normal bleeding tendency in mice. Altogether, these results suggest that a direct application of CDOTs may contribute to the development of new antiplatelet drugs for the treatment of arterial thromboembolic diseases [9]. 

In the application of graphene nanomaterials for dental regenerative engineering, Di Carlo et al. proposed the covalent functionalization of a graphene oxide (GO)-decorated cortical membrane (Lamina^®^) in the promotion of the adhesion, growth and osteogenic differentiation of DPSCs (Dental Pulp Stem Cells). The GO-decorated Laminas demonstrated an increase in the roughness of Laminas and a reduction in toxicity and did not affect the membrane integrity of DPSCs. In conclusion, this study showed that the GO covalent functionalization of Laminas was effective; was relatively easy to obtain; and favored both the proliferation rate of DPSCs, probably due to the capacity of GO to adsorb proteins present in the medium, and the deposition of calcium phosphate. Overall, this study is promising because the proposed material holds potential as a useful substrate in facilitating in vivo bone regeneration [10]. 

In antibacterial applications, Nicosia et al. synthesized novel NanoHybrid Systems based on graphene, polymers and AgNPs (namely, NanoHy-GPS) using an easy microwave irradiation approach free of reductants and surfactants. The fine-tuned hybrid system combines the properties of polymers, graphene and AgNPs as a potential on-demand antimicrobial coating system. Polymers play key roles in ensuring the coating compatibility of the graphene platform, making it adaptable for a specific substrate. The driving force of this strategy is the tuning of the interfacial interactions towards targeted substrates, thus optimizing the homogeneity of the dispersion of the GO derivatives within specific polymer matrices. The formulation of functionalized graphene with AgNPs entrapped in suitable polymers resulted in a doubly beneficial effect: an increase in graphene processability and achievement of graphene-enriched antimicrobial nanobiomaterials. NanoHy-GPS was proposed as a potential alternative to common antibacterial agents, which leak into the environment and/or within organisms’ tissues [11]. 

Finally, Cordaro et al. proposed a review aimed at providing a comprehensive and exhaustive summary of the contributions of graphene-based nanomaterials to liquid biopsy. Liquid biopsy is considered an innovative method that has provided surprising perspectives in the early diagnosis of severe diseases such as cancer, metabolic syndrome, and autoimmune and neurodegenerative disorders and in monitoring their treatment. Although nanotechnology based on graphene has been poorly applied for the rapid diagnosis of viral diseases, the extraordinary features of graphene (i.e., high electronic conductivity, large specific area and surface functionalization) can also be exploited for the diagnosis of emerging viral diseases, such as coronavirus disease 2019 (COVID-19) [12]. 

The variety of applications covered by the nine articles published in this Special Issue of *Nanomaterials* “Carbon Nanomaterials for Therapy, Diagnosis and Biosensing” is proof of the growing attention on the use of carbon nanomaterials in the biomedical/pharmaceutical field in recent years. We hope that the readers enjoy reading these articles and find them useful for their research and for advancing carbon nanomaterials from the laboratory to clinical nanomedicine. Finally, we acknowledge all of the authors who contributed their work to this Special Issue as well as the editorial board of *Nanomaterials* for all of their support.
